# Supplementary material for: Platelet Microparticles Decrease Daunorubicin-Induced DNA Damage and Modulate Intrinsic Apoptosis in THP-1 Cells
Source: Int J Mol Sci. 2021 Jul 6;22(14):7264. doi: 10.3390/ijms22147264 (PMC8304976; doi:10.3390/ijms22147264)
Supplement: Supplementary file 1 [file ijms-22-07264-s001.zip › ijms-1260781-supplementary.pdf]

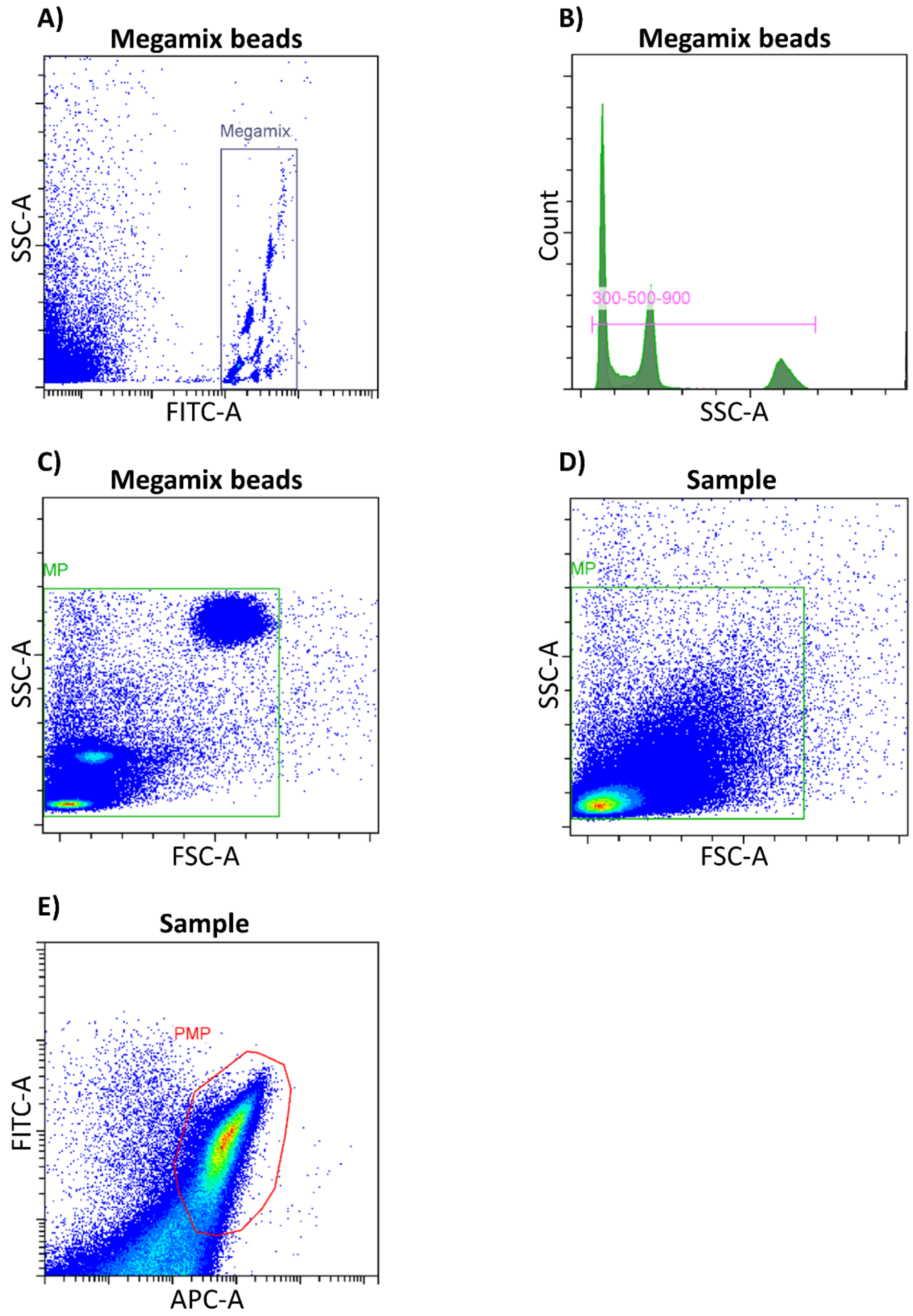

Figure S1. Gating strategy for flow cytometric platelet microparticle (PMP) quantitation. (**A**) Megamix beads gate was first established in a FITC-A vs. SSC-A plot using Megamix Plus FSC beads, including all doublets. (**B**) The three bead populations, 0.3, 0.5, and 0.9 µm, were then visualized in a SSC-A histogram of the "Megamix" gate, establishing the true range of SSC-A for the beads while discriminating doublets. (**C**) Microparticle gate was then established in a FSC-A vs. SSC-A plot. (**D**) Sample was acquired and gated as in (C). (**D**) PMPs were identified in an annexin V FITC-A vs. CD61 APC-A plot, including only hi/hi events in the microparticle gate. MP, microparticles

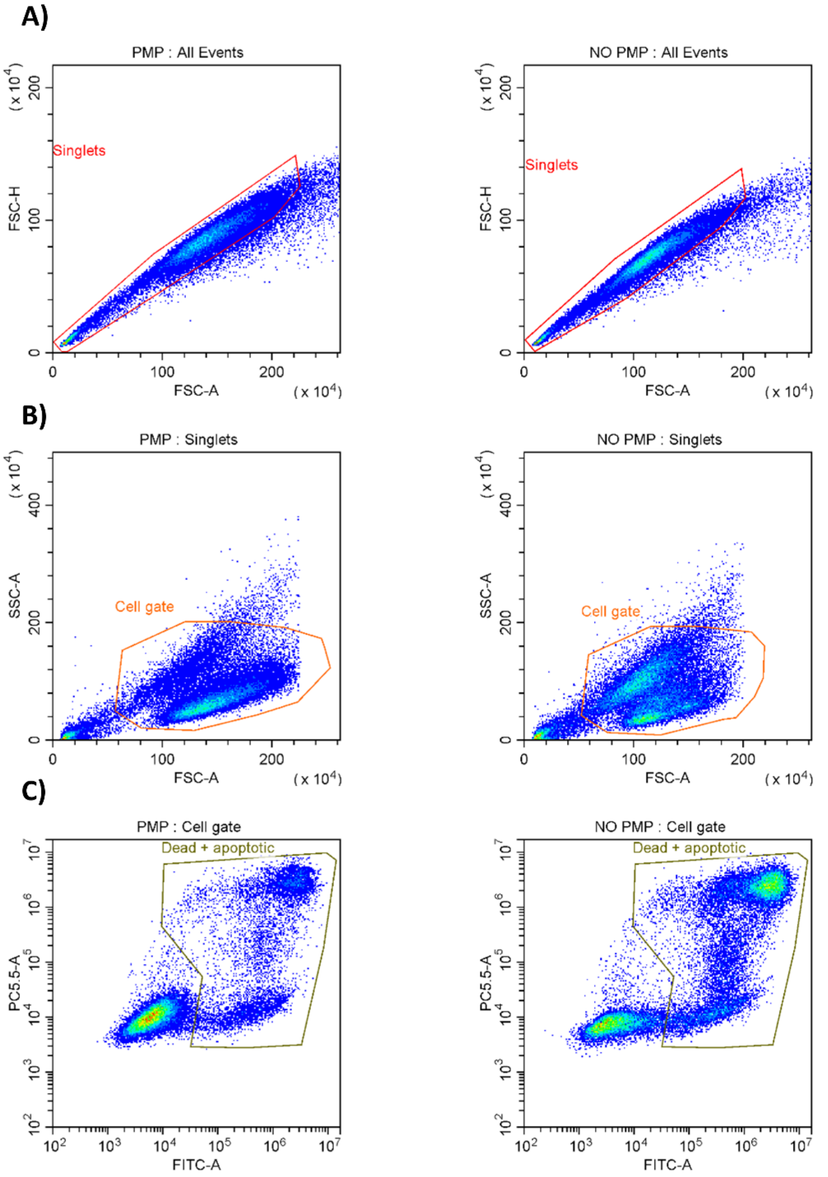

Figure S2. Gating strategy for apoptosis assay. (**A**) Doublets were discriminated in a FSC-A vs. FSC-H plot. (**B**) “Cell gate” was then defined in a FSC-A vs. SSC-A plot. (**C**) Dead and apoptotic cells were finally identified in an annexin V FITC vs. propidium iodide plot. Sample from the experiments with alantolactone. A similar strategy to define “Cell gate” was used for the caspase assays.

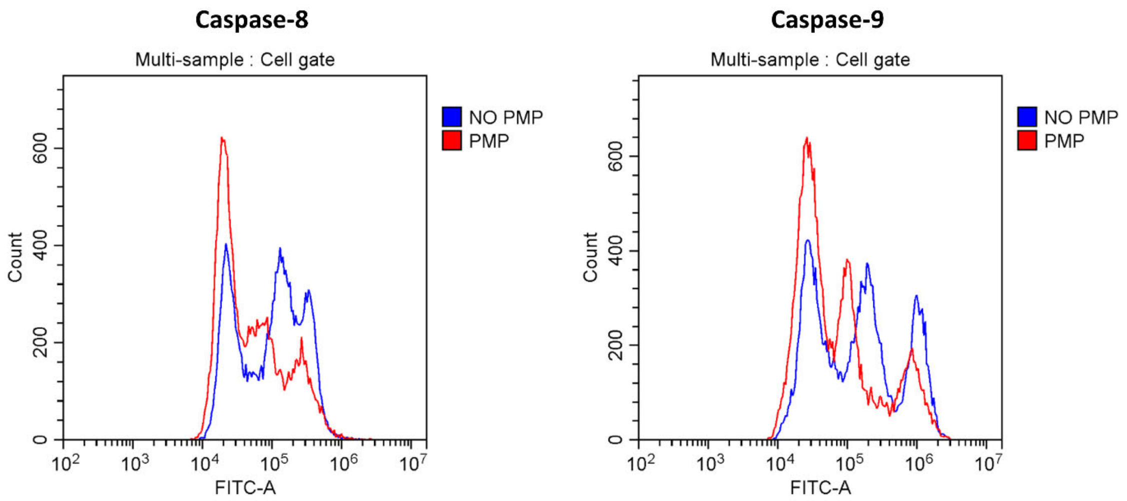

Figure S3. Supplementary histogram overlays for the data in Fig. 2.

Table S1. List of apoptosis inductors

Substance	Supplier	Solvent	Final concentration	Incubation time (hours)
Daunorubicin	Sigma Aldrich	dH20	0.5 µM	24
Alantolactone	Sigma Aldrich	DMSO	20 µM	24
Staurosporine	Sigma Aldrich	DMSO	1 µM	6
MG 132	Sigma Aldrich	DMSO	2.5 µM and 5 µM	24
TRAIL	Sigma Aldrich	dH20	50 ng/mL	24
Piceatannol	Sigma Aldrich	DMSO	50 µM	24

Table S2. List of antibodies.

Primary antibody	Host	Clone	Supplier	Dilution	Incubation time	Incubation temperature
BAK1	Mouse	4C2	Thermo Fisher	1:50	30 min	Room temp.
BCL2	Mouse	100/D5	Thermo Fisher	1:40	60 min	Room temp.
BMF	Rat	12E10	Thermo Fisher	1:100	60 min	Room temp.
MCL1	Mouse	OTI2E11	Thermo Fisher	1:100	60 min	Room temp.
PUMA	Rabbit	SR42-09	Thermo Fisher	1:100	30 min	Room temp.

Secondary antibody	Host	Species reactivity	Conjugate	Supplier	Dilution	Incubation time	Incubation temperature
Goat anti-Mouse IgG (H+L)	Goat	Mouse	DyLight 488	Thermo Fisher	1:25	30 min	Room temp.
Goat anti-Rabbit IgG (H+L)	Goat	Rabbit	AlexaFluor 488	Thermo Fisher	1:200	30 min	Room temp.
Goat anti-Rat IgG (H+L)	Goat	Rat	AlexaFluor 488	Thermo Fisher	1:200	30 min	Room temp.
